# Zscan4 is expressed specifically during late meiotic prophase in both spermatogenesis and oogenesis

**DOI:** 10.1007/s11626-016-0096-z

**Published:** 2016-10-03

**Authors:** Kei-ichiro Ishiguro, Manuela Monti, Tomohiko Akiyama, Hiromi Kimura, Nana Chikazawa-Nohtomi, Miki Sakota, Saeko Sato, Carlo Alberto Redi, Shigeru B. H. Ko, Minoru S. H. Ko

**Affiliations:** 1 Department of Systems Medicine, Keio University School of Medicine, 35 Shinanomachi, Shinjuku, Tokyo, 160-8582 Japan; 2 Research Center for Regenerative Medicine, Fondazione IRCCS Policlinico San Matteo, viale Golgi 19, 27100 Pavia, Italy; 3 Dipartimento di Biologia e Biotecnologie L. Spallanzani, University of Pavia, via A. Ferrata 9, 27100 Pavia, Italy; 4 Institute of Molecular Embryology and Genetics, Kumamoto University, 2-2-1 Honjo, Chuo-ku, Kumamoto, 860-0811 Japan

**Keywords:** GV oocyte, SN and NSN oocyte, Ovary, Testis, ES cell, Preimplantation embryo

## Abstract

Mouse zinc finger and SCAN domain containing 4 (Zscan4) proteins, which are encoded by multiple copies of *Zscan4* genes, are expressed specifically in preimplantation embryos in vivo and embryonic stem (ES) cells in vitro. However, the expression patterns of mouse Zscan4 in vivo have been largely elusive. Here, we show that Zscan4 proteins are expressed in adult ovaries and testes. In ovaries, Zscan4 proteins were detected in germinal vesicle (GV) stage oocytes in antral follicles, indicating that *Zscan4* genes are activated during the diplotene/dictyate stage in meiotic prophase I. Remarkably, Zscan4 showed different spatial localization patterns between two distinct GV oocytes, which can be distinguished by global chromatin organization—surrounded nucleolus (SN) and non-surrounded nucleolus (NSN). These spatiotemporal differences in Zscan4 localizations correlated with the transition of RNA polymerase II-mediated transcriptional status during GV oocyte maturation. In testes, Zscan4 proteins were detected in spermatocytes at late pachytene/diplotene stages and in Sertoli cells. These results suggest that Zscan4 may play critical roles during late meiotic prophase in both males and females.

**Electronic supplementary material:**

The online version of this article (doi:10.1007/s11626-016-0096-z) contains supplementary material, which is available to authorized users.

## Introduction

In the mouse genome, nine copies of zinc finger and SCAN domain containing 4 (*Zscan4*) genes are encoded in the *Zscan4* locus: six members of *Zscan4* genes (*Zscan4a*, *Zscan4b*, *Zscan4c*, *Zscan4d*, *Zscan4e*, and *Zscan4f*) and three pseudogenes (*Zscan4-ps1*, *Zscan4-ps2*, and *Zscan4-ps3*) (Falco et al. 2007). Because it is difficult to distinguish between the copies of the *Zscan4* genes and since there is only a single copy of *ZSCAN4* in the human genome (Falco et al. 2007), the mouse Zscan4 genes are collectively called *Zscan4* (Falco et al. 2007; Zalzman et al. 2010; Amano et al. 2013). The mouse *Zscan4* genes were originally identified for their unique expression during zygotic genome activation (ZGA) in late two-cell stage embryos, but they are also expressed in mouse embryonic stem (ES) cells (Falco et al. 2007; Akiyama et al. 2015).

In mouse ES cells, the transcription of *Zscan4* is transient and reversible, resulting in a small population (1–5%) of Zscan4+ cells in culture at a given time point (Zalzman et al. 2010). A burst of *Zscan4* transcription is accompanied by unique biological events, including transient expression of other ZGA-specific genes (Akiyama et al. 2015), rapid derepression and rerepression of heterochromatin regions (Akiyama et al. 2015), rapid telomere extension (Zalzman et al. 2010), and blockage of global translation (Hung et al. 2013). Moreover, Zscan4 has been shown to enhance the efficiency of generating mouse induced pluripotent stem (iPS) cells and their quality (Hirata et al. 2012; Jiang et al. 2013). These data suggest that Zscan4 plays critical biological roles in ES cells.

Our previous studies by in situ hybridization and transcriptome analyses showed that *Zscan4* messenger RNA (mRNA) is expressed in mouse preimplantation embryos with the peak at the late two-cell stage, although its protein level is yet to be analyzed (Falco et al. 2007; Zalzman et al. 2010). In accordance with those observations, it was demonstrated that knockdown of *Zscan4* by siRNA leads to delayed progression from the two-cell to four-cell stage and consequently implantation failure (Falco et al. 2007)*.* Thus, *Zscan4* is expressed transiently in preimplantation embryos in vivo, as has been shown in ES cells in vitro. However, a precise expression pattern of *Zscan4* in vivo has been largely elusive, which could be due to the transient nature of expression in a small subset of cell lineages.

In this study, we examined the expression of Zscan4 protein in mouse tissues in vivo. Our cytological analysis demonstrated that Zscan4 protein is expressed in adult reproductive organs—ovary and testis—in addition to preimplantation embryos. Intriguingly, our data suggest that the spatiotemporal expression pattern of Zscan4 protein correlates with the transition of chromatin reorganization and accompanying RNA polymerase II-mediated transcriptional status during oocyte maturation. Moreover, we found that Zscan4 protein is expressed during late meiotic prophase I in adult ovaries and testes. Thus, our current observations will shed light on further approaches to study critical biological functions of Zscan4 in both male and female reproductive cell lineages in vivo.

## Materials and Methods

### ES cell culture

The derivative cells from MC1 (Zalzman et al. 2010; Akiyama et al. 2015) or TA1 mouse ES cells (F1 hybrid of C57BL/6J × 129S6/SvEvTac) (Amano et al. 2013) were used. *Emerald* green fluorescent protein (GFP) knock-in (Z4EmKI) ES cells were described in the accompanied paper (Ishiguro et al. 2016). ES cell lines were maintained on gelatin-coated, feeder-free plates in complete ES medium (Zalzman et al. 2010).

### Animal experiments

Animal experiments were approved by the Institutional Animal Care and Use Committee (approval nos. 12702-0, 24-010-11).

### FACS

The fluorescent intensity analysis and sorting of Emerald of Z4c-Em ES cells were performed using a BD FACSAria II. The cells were sorted according to the fluorescent intensity of Emerald and collected into mouse ES cell culture medium at 4°C.

### Production of antibodies

Polyclonal antibodies against mouse Zscan4 (a.a.1-506) were produced by inserting its complementary DNA (cDNA) fragment in-frame with pET19b (Merck Millipore , Tokyo Japan) in *E. coli* strain BL21-CodonPlus(DE3). His-tagged recombinant proteins were solubilized in a denaturing buffer (6 M HCl-Guanidine, 20 mM Tris-HCl [pH 7.5]) from the inclusion body and purified by Ni-NTA (QIAGEN, Tokyo Japan) under denaturing conditions. After dialyzing against PBS, the purified protein was used to immunize rats and rabbits. The antibodies were affinity purified from the immunized crude serum with immobilized antigen on CNBr-activated Sepharose (GE Healthcare, Tokyo Japan).

### Antibodies

The following antibodies were used: mouse anti-GFP (Santa Cruz, Tokyo Japan : sc-9996), rabbit anti-GFP (Abcam, Tokyo Japan : ab6556), rabbit anti-RNA polymerase II CTD repeat (phospho S5) (Abcam: ab5131), rabbit anti-RNA polymerase II CTD repeat (phospho S2) (Abcam: ab5095), mouse anti-KAP1 (Abcam: ab22553), mouse anti-SYCP3 (Ishiguro et al. 2011), and human anti-centromere antigen (Inova Diagnostics, San Diego CA).

### Histological analysis

Ovaries from 4- to 8-wk-old mice and testes from 13-18 dpc embryos and 8-wk-old mice were embedded in Tissue-Tek O.C.T. compound (Sakura Finetek, Tokyo Japan) and frozen in liquid nitrogen. Cryosections were prepared on the MAS-coated slides (Matsunami, Osaka Japan) at 8 μm thickness and then air dried and fixed in 4% paraformaldehyde in PBS at pH 7.4.

### Immunofluorescence staining

ES cells were grown on a cover glass and fixed in 4% paraformaldehyde (PFA) for 5 min at room temperature and permeabilized in 0.1% TritonX100 in PBS for 10 min. The cells were blocked for 10 min in PBS, 3% BSA, and incubated at room temperature with the primary antibodies in a blocking solution. After three washes in PBS, the cells were incubated for 1 h at room temperature with Alexa-dye-conjugated secondary antibodies (1:1000; Invitrogen) in a blocking solution. DNA was counterstained with Vectashield mounting medium containing 4′,6-diamidino-2-phenylindole (DAPI) (Vector Laboratory, Burlingame CA).

GV, metaphase I (MI), and metaphase II (MII) oocytes and preimplantation embryos were fixed in 4% PFA/PBS for 30 min at room temperature, washed with PBS, and permeabilized with PBS +0.1% Triton X-100. After a brief wash in PBS, they were blocked in 5% BSA/PBS for 30 min at room temperature and then immunostained with first antibodies diluted 1:500 in blocking solution at 4°C overnight. Samples were then briefly washed in PBS and incubated with Alexa-dye-conjugated second antibodies diluted 1:1000 in blocking solution for 1 h at room temperature. Samples were then mounted on slides with Vectashield with DAPI (Vector Laboratories). The same protocol has been used for Zscan4 immunofluorescence on serial sections of adult ovaries and testes with the exclusion of the permeabilization step.

### Imaging

Immunostaining images were captured with DeltaVision and processed with DeltaVision SoftWoRx software (GE Healthcare) or confocal microscope FluoView Fv10i (Olympus, Tokyo Japan) and processed with FluoView Software. All images shown were Z-stacked. GFP fluorescence and bright field images were captured with OLYMPUS IX73 fluorescence microscope and processed with CellSens standard program.

The intensity profile of Zscan4 signal in both surrounded nucleolus (SN) and non-surrounded nucleolus (NSN) oocytes has been obtained by selecting the region of interest (nucleolus) using the 2D tool of the cited software (Monti and Redi 2016). Each oocyte has been analyzed with Image J to count the corrected total cell fluorescence (CTCF). Briefly, each oocyte belonging to the three groups analyzed (SN spotty, SN diffuse, and NSN) has been selected and the area, integrated density and mean gray value, measured. In the meantime, a region next to each oocyte with no fluorescence has been measured as a background. The CTCF has been obtained using the following formula: Integrated density − (area of selected cell × mean fluorescence of backgrounds).

### In vitro germinal vesicle oocyte culture and maturation

Ovaries collected from 6- to 8-wk-old female mice were used after 46 h of treatment with 5 IU of pregnant mare serum gonadotropin (PMSG, Sigma, Tokyo Japan). GV oocytes were isolated by puncturing the follicles in KSOM (Merck Millipore , Tokyo Japan) containing 250 μM 3-isobutyl-1-metylxanthine (IBMX, Sigma). To induce resumption of meiosis, the oocytes were cultured in KSOM at 37°C after withdrawing IBMX. Oocytes that had not undergone GV breakdown (GVBD) by 90 min were removed from the experiment.

For in vitro maturation of GV oocytes, 4- to 6-wk-old females were used after 46 h of treatment with 5 IU of PSMG. Briefly, antral oocytes were collected by puncturing the antral follicles, washed in M2 medium, and stained with the fluorochrome Hoechst 33342 to distinguish chromatin organization in the NSN and SN types, as fully described elsewhere (Monti and Redi 2016). After sorting, SN and NSN oocytes were cultured in M2 medium at 37°C and 5% CO_2_ overnight. Subsequent MII-SN and MII-NSN oocytes were fixed in PFA 4% for 30 min at room temperature and washed with PBS, blocked with 3% BSA/PBS containing 0.1% Triton X-100 for 1 h at room temperature, and then immunostained as described above.

### Collection and in vitro culture of preimplantation embryo

Female mice were injected with 5 U of pregnant mare serum gonadotropin (PMSG; Merck Millipore , Tokyo Japan) and 48 h later with 5 U of hCG (Calbiochem), and then crossed with a male mouse. Embryos were flushed out of the mouse oviducts and cultured in a drop of KSOM (Millipore).

### Reverse transcription polymerase chain reaction

Total RNA was isolated from tissues, isolated oocytes, and ES cells using TRIzol (Thermo Fisher, Yokohama Japan). cDNA was generated from total RNA using Superscript III (Invitrogen) followed by PCR amplification using Ex-Taq polymerase (Takara, Shiga Japan) and template cDNA. Sequences of primers used to generate RT-PCR products from cDNA are as follows:

SYCE1-787F: 5′-cacgagcagctgcagcagaggtgc-3′.

SYCE1-990R: 5′-ttaggtcctgcttgatgggcgctc-3′.

Sycp3-368F: 5′-gctgacatcaacaaagctcttcttg-3′.

Sycp3-605R: 5′-gttgttgtcgaaaaagattagatag-3′.

MuERVL-F: 5′-CCATCCCTGTCATTGCTCA-3′.

MuERVL-R: 5′-CCTTTTCCACCCCTTGATT-3′.

GAPDH-F: 5′-ttcaccaccatggagaaggc-3′.

GAPDH-R: 5′-ggcatggactgtggtcatga-3′.

Zscan4F: 5′-cagatgccagtagacaccac-3′.

Zscan4R: 5′-gtagatgttccttgacttgc-3′.

Meikin-F: 5′-agatggacagcttgttgtcgagta-3′.

Meikin-R: 5′-ctcagcaaatacaacctcagaagc-3′.

GFP-F: 5′-ACGTAAACGGCCACAAGTTC-3′.

GFP-R: 5′-AAGTCGTGCTGCTTCATGTG-3′.

## Results

### Mouse *Zscan4* mRNA is expressed in the adult testis and ovary

**Figure 1.**
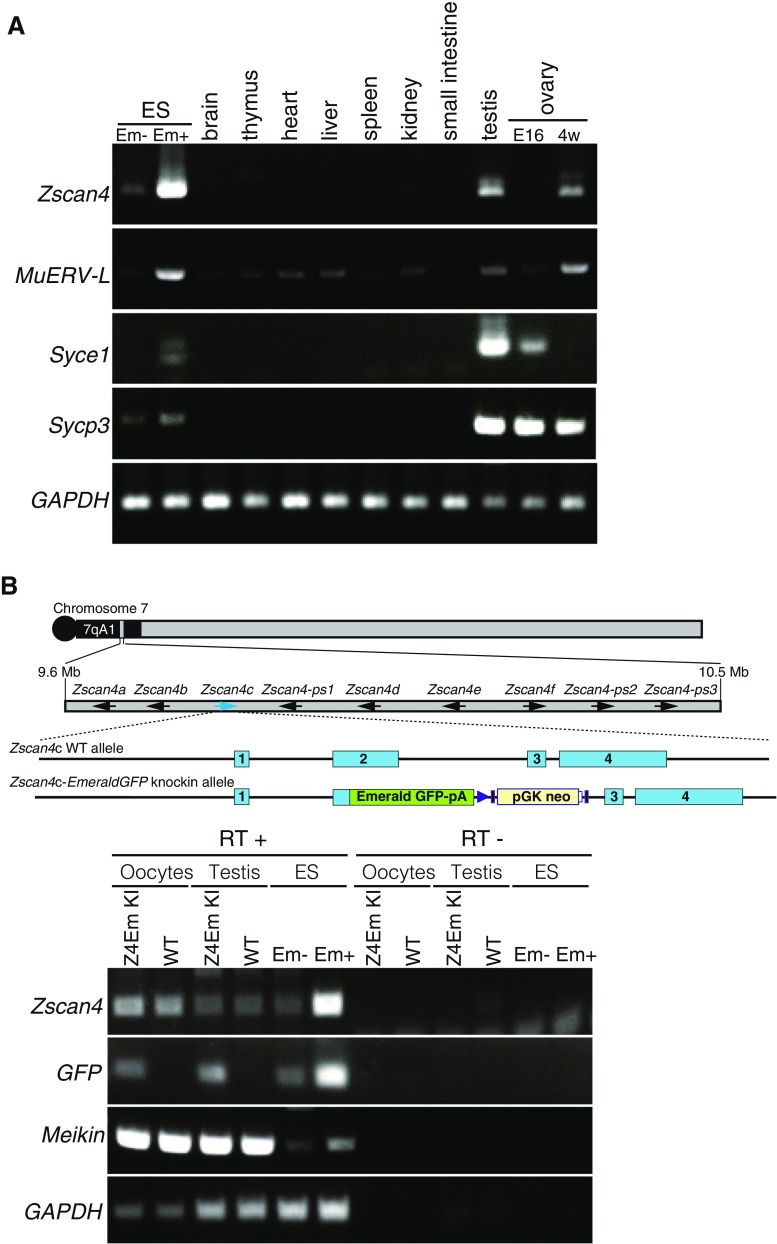
*Zscan4* mRNA expression in the testis and ovary. (*A*) Tissue specificity of mRNA expression for the indicated genes was analyzed by RT-PCR. *E16* fetal ovary at embryonic day 16; *4w* 4-wk ovary; *Em− and Em+* Emerald GFP negative and positive populations of mouse MC1 ES cells enriched by FACS, respectively; *Syce1* and *Sycp3*: meiosis specific markers. *MuERV-L* retrotransposon supposed to be highly expressed in Em+ ES cell. (*B*) Expression of endogenous *Zscan4* and exogenous *Emerald GFP*, which is knocked-in at the *Zscan4c* locus, was analyzed by RT-PCR. RNA was extracted from the isolated oocytes and testis in *Emerald GFP* knock-in (Z4EmKI) and wild-type mice and from Z4EmKI ES cells. Schematic Z4EmKI locus is shown (*upper*). *Em− and Em+* Emerald negative and positive enriched populations of Z4EmKI ES cells, respectively. *Meikin* meiosis I specific marker (Kim et al. 2015). *RT−* control PCR without reverse transcription.

It has been shown that *Zscan4* genes are expressed in mouse late two-cell stage preimplantation embryos and only in 1–5% of ES cells at a given time point (Falco et al. 2007; Zalzman et al. 2010). Because *Zscan4* expression in vivo has been largely elusive, we examined its expression pattern in mouse tissues by RT-PCR (Fig. 1 *A*). Expression of *Zscan4* mRNA was detected in the adult ovary and testis, although the levels in these tissues were modest compared to a positive control—Emerald-positive (Em+) MC1 ES cells in which the *Emerald GFP* transgene is expressed under *Zscan4c* promoter (Zalzman et al. 2010). Notably, *Zscan4* mRNA was detected in adult but not embryonic ovary, suggesting that *Zscan4* is expressed in oocytes at later than the diplotene/dictyate stage of meiotic prophase I or in a specific cell type associated with folliculogenesis. Because the RT-PCR in this study cannot distinguish individual *Zscan4* genes and pseudogenes (*Zscan4a*, *b*, *c*, *d*, *e*, *f*, *ps1*, *ps2*, *ps3*), the observed expression level represents total transcripts potentially contributed from individual *Zscan4* loci in the testis and ovary, as has been shown in ES cells (Akiyama et al. 2015). The activation of the endogenous *Zscan4* locus was further validated by the demonstration of the expression of *Emerald* in oocytes and testes isolated from *Emerald*-knock-in (Z4EmKI) mice, in which *Emerald* was inserted into the endogenous *Zscan4c* locus (see accompanied manuscript, Ishiguro et al.) (Fig. 1 *B*).

### Presence of Zscan4 proteins in germinal vesicle oocytes and preimplantation embryos

The aforementioned results led us to examine Zscan4 protein expression in mouse tissues. To this end, we generated new polyclonal antibodies against the full length (506 a.a.) of the Zscan4 protein (see accompanied manuscript), because our previous anti-Zscan4 antibody, which was raised against the most C-terminal-peptide (14 a.a.) epitope of Zscan4c (Zalzman et al. 2010), might have missed detection of truncated forms: Zscan4a, Zscan4b, and Zscan4e proteins.

**Figure 2.**
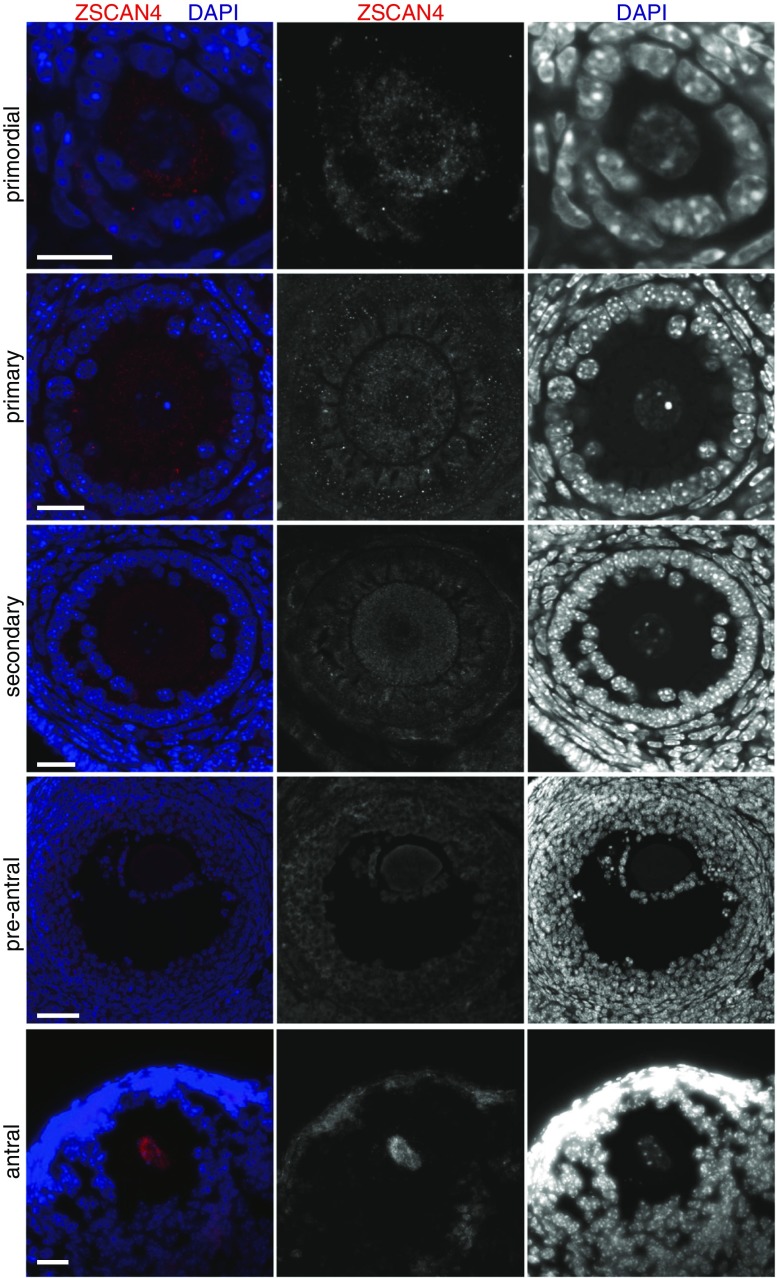
Zscan4 protein is expressed in oocytes of antral follicles. Ovary sections were immunostained as indicated, showing Z-stack sections of Zscan4 immunofluorescence of primordial, primary, secondary, preantral, and antral follicles. *Scale bars*, 20 μm.

Immunostaining of adult ovary sections showed background-level signals in ovarian somatic cells and primordial, primary, secondary, and preantral oocytes (Fig. 2). By contrast, Zscan4 protein was detected in the nuclei of oocytes residing in the antral follicles (Fig. 2). These results were consistent with the *Zscan4* mRNA expression in oocytes isolated from the adult ovary (Fig. 1 *B*) but not the embryonic ovary (Fig. 1 *A*), suggesting that Zscan4 starts to be expressed in or after diplotene/dictyate stages but not before these stages.

**Figure 3.**
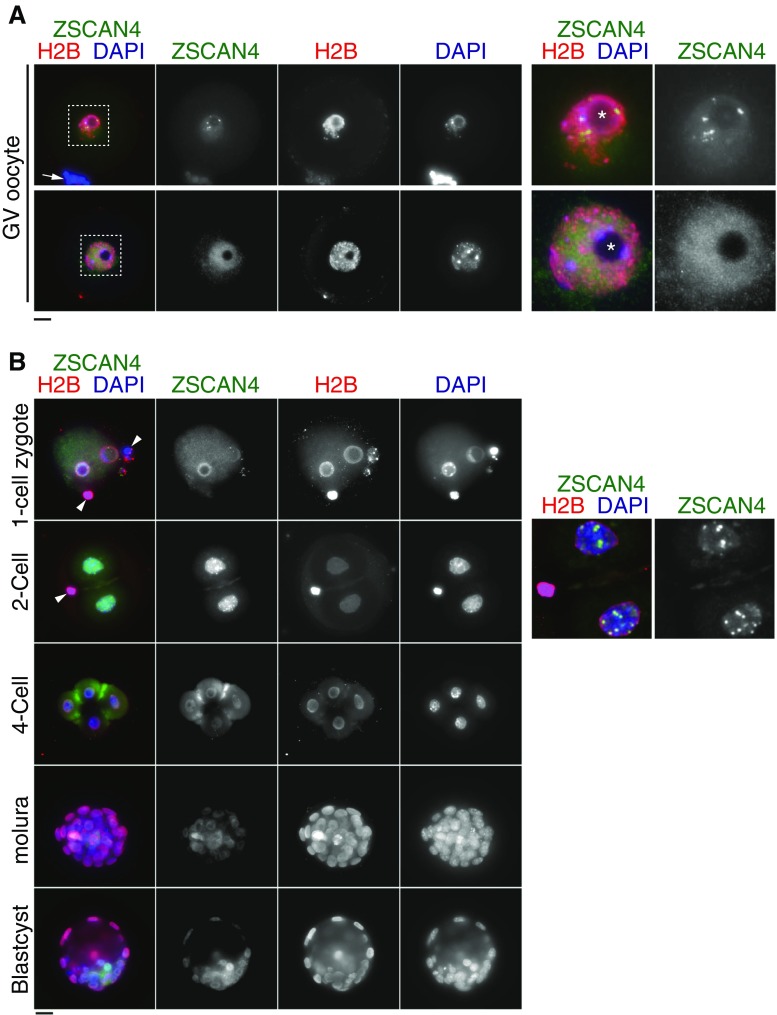
Zscan4 protein is expressed in GV oocytes and preimplantation embryos. (*A*) GV oocytes were immunostained as indicated. Two types of immunostaining patterns in GV oocytes are shown. The *upper example* shows the spotty immunostaining pattern of Zscan4, some of which surround a nucleolus. The *lower example* shows the faint diffusive immunostaining pattern of Zscan4. *Enlarged images* of the nuclei are shown on the *right*. *Arrow* indicates cumulus granulosa cells associated with zona pellucida of GV oocyte. *Asterisk* indicates nucleolus. (*B*) Preimplantation embryos at different developmental stages were immunostained as indicated. Enlarged deconvolution images of partial Z projection for two cells are shown on the *right*, emphasizing intense Zscan4 foci. *Arrowhead* indicates polar body in two cells. *Scale bars*, 20 μm.

To examine Zscan4 protein expression in more detail, we used isolated GV oocytes and embryos at the subsequent stages. Interestingly, GV oocytes showed two types of Zscan4 immunostaining patterns: spotty signals around the nucleolus and a more diffuse signal dispersed through the nucleus (Fig. 3 *A*). This finding will be further elaborated in the section below. After germinal vesicle break down (GVBD) followed by resumption of meiosis I, Zscan4 seemed to disappear from the chromosome, though a trace of the protein may remain (Supplementary Fig. 1A). After fertilization at the one-cell stage, weak immunostaining signals were observed in the periphery of pronuclei, which were overlapped with histone 2B (H2B) staining. Because Zscan4 mRNAs were not detected at this stage, the trace amount of Zscan4 proteins may be brought in from gametes. At the two-cell stage, Zscan4 proteins robustly culminated with some intense spots in the nuclei (Fig. 3 *B*), consistent with a sharp increase of *Zscan4* mRNAs detected by in situ hybridization and transcriptome studies (Hamatani et al. 2004; Falco et al. 2007; Macfarlan et al. 2012). After the two-cell stage, Zscan4 protein levels decreased precipitously. Taken together, Zscan4 proteins were produced and present in GV oocytes (diplotene/dictyate meiotic stages) and two-cell embryos.

### Two distinct patterns of Zscan4 localizations in germinal vesicle oocytes

**Figure 4.**
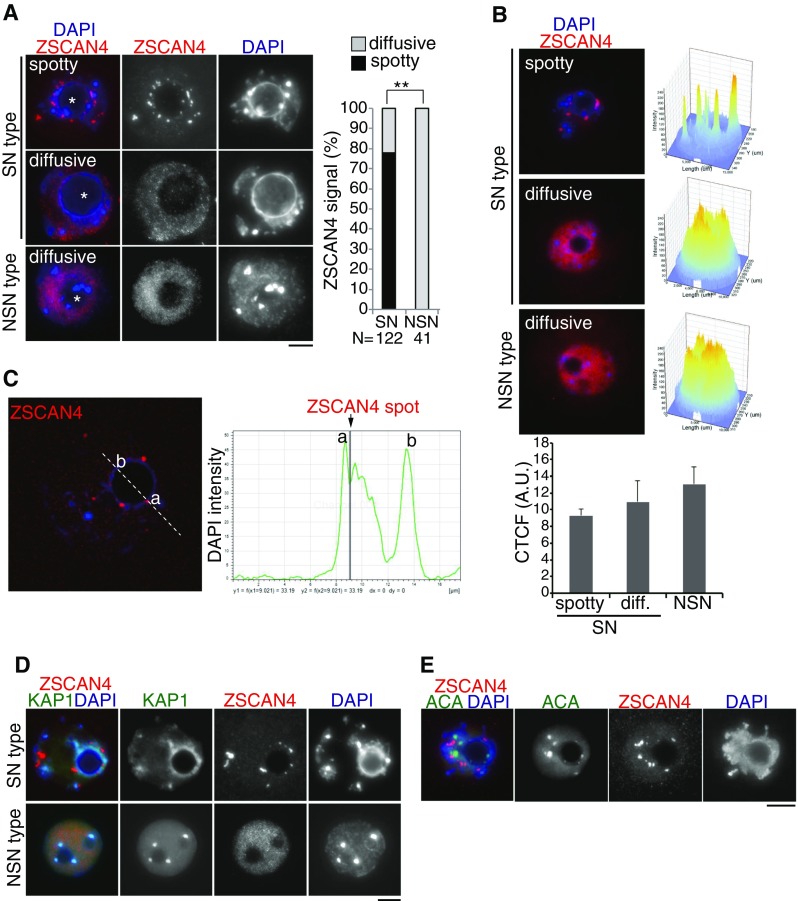
Zscan4 localization in SN- and NSN-type GV oocytes. (*A*) GV oocytes, classified as SN type and NSN type by heterochromatin morphology relative to nucleolus (shown by asterisk), were immunostained as indicated. The spatial localization pattern of Zscan4 was classified as spotty (limited number of foci) and diffusive (dispersed in nuclei). The immunostaining pattern of Zscan4 is quantified in the *right graph*. The number of analyzed GV oocytes (pooled from four independent experiments) is indicated. **P < 0.001 (Pearson’s chi-square test). (*B*) Intensity profile of Zscan4 immunostaining signal obtained with the FluoView Software from representative GV oocytes with a SN spotty (*top*, *n* = 21), SN diffuse (*middle*, *n* = 11), and NSN (*bottom*, *n* = 23). The value of pixel intensity on *Z*-axis is equal to 200 for the spotty signal (SN spotty) and to 180 (SN diffuse, NSN) for the oocytes with diffuse signal as inferable from the picture. Corrected total cell fluorescence (CTCF, arbitrary units) is shown in the *bottom graph* with SEM. *C* Zscan4 and DAPI signals were scanned across a nucleolus in SN-type GV oocyte (*dashed line*). Signal intensity of DAPI (*green*) and Zscan4 (*gray bar*) is shown on the bottom. *D* SN-type and NSN-type GV oocytes were immunostained for KAP1: a heterochromatic protein which is potentially associated with Zscan4. *E* SN-type GV oocyte was immunostained for anti-centromere antigen (ACA). *Scale bars*, 10 μm.

As mentioned above, detection of two types of Zscan4 immunostaining patterns in isolated GV oocytes (Fig. 3 *A*) prompted us to examine this finding in more detail. We first noticed that these two immunostaining patterns correlated well with two distinct DAPI-staining patterns: a ring of DAPI-positive heterochromatin around the nucleolus (surrounded nucleolus [SN]) or a more diffuse DAPI signal around the nucleolus (non-surrounded nucleolus [NSN]) (Debey et al. 1993; Zuccotti et al. 1995), which are known to correlate with competency of resuming meiosis I and developmental potential of embryos (Inoue et al. 2008; Monti et al. 2013). Indeed, 78% of SN-type GV oocytes showed a spotty Zscan4 signal, whereas the remaining 22% showed a dispersed Zscan4 pattern. Consistency of the spotty Zscan4 immunostaining pattern was confirmed by overlapped immunostaining with two additional anti-Zscan4 antibodies raised in rat and rabbit (Supplementary Fig. 2). Also, the intensity profile measurement of the immunostaining signals in GV oocytes with NSN- and SN-Zscan4 diffuse pattern versus SN-Zscan4 spotty pattern clearly indicated a comparable level of immunostaining signals among the samples (Fig. 4 *B*), suggesting that the distribution, but not the quantity, of Zscan4 proteins are different in these GV oocytes.

More detailed profiling of signal intensity for Zscan4 immunostaining and DAPI staining across a nucleolus indicated that Zscan4 foci resided at a DAPI-dense region rather than the inner side of the nucleolus in SN-type GV oocytes (Fig. 4 *C*). This notion was further supported by the 3D reconstruction of immunostaining images (Supplementary Movies 1, 2). In mice, most constitutive heterochromatin domains are associated with pericentromeric regions and organized into chromocenters (Saksouk et al. 2015). Previously, we have shown the involvement of Zscan4 in heterochromatin regulation in mouse ES cells (Akiyama et al. 2015). Especially, mass spectrometry analyses of protein complexes associated with exogenous FLAG-tagged Zscan4 (Akiyama et al. 2015) and endogenous Zscan4 (see accompanied manuscript) revealed Zscan4’s association with transcriptional repressors, KDM1A/LSD1, KAP1/TIF1β, and HDAC1. Therefore, we asked whether in SN-type GV oocytes Zscan4 accumulates to heterochromatin regions by co-immunostaining with KAP1 or centromeric protein (Fig. 4 *D*, *E*) (Supplementary Movie 3). Although Zscan4 foci did not seem to be overlapped with centromeric or pericentromeric regions, they closely coincided with KAP1-stained heterochromatin regions surrounding a nucleolus in SN-type GV oocytes. Thus, it is possible that Zscan4 preferentially localizes to specific sites in the heterochromatin region in SN-type GV oocytes.

### Zscan4 localization patterns correlate with transcription status in germinal vesicle oocytes

**Figure 5.**
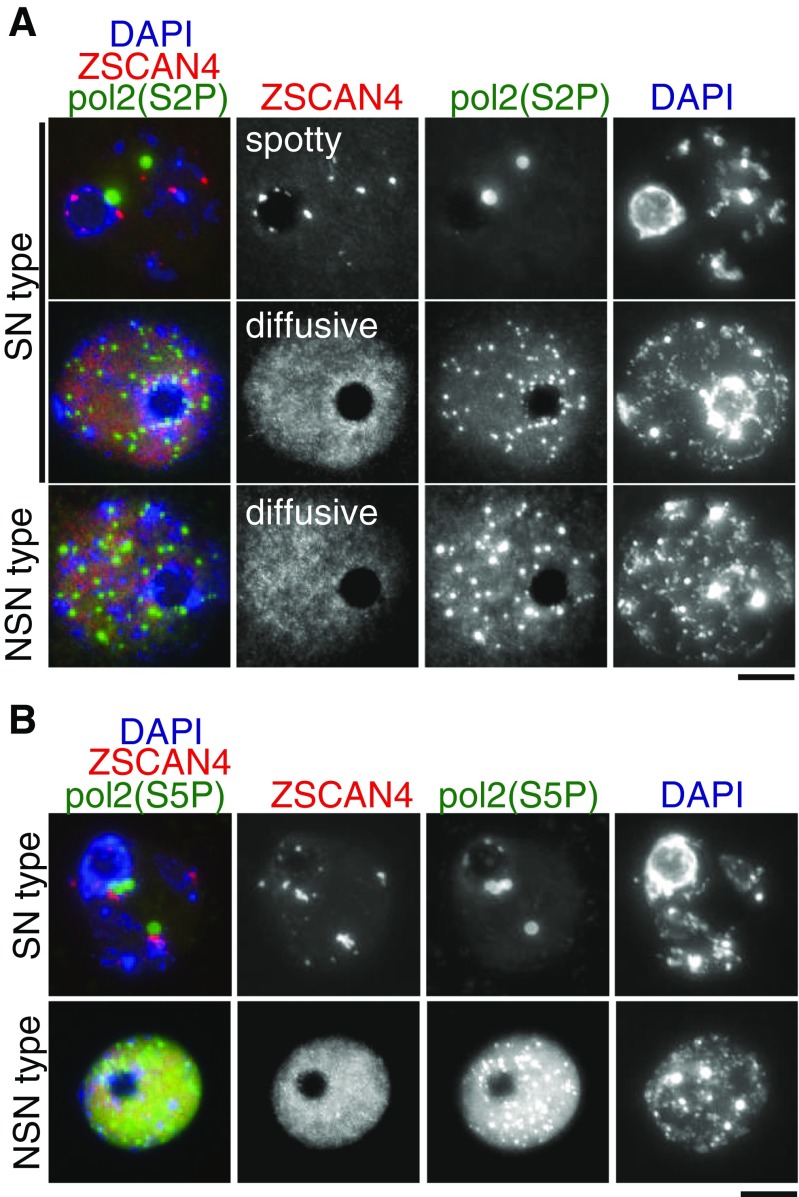
Localization patterns of Zscan4 correlate with Pol II-mediated transcription activity in GV oocytes. (*A*) SN- and NSN-type GV oocytes were immunostained for *Pol2(S2P)*: RNA polymerase II CTD repeat (phospholylated Ser2), a marker for active transcriptional elongation. Weak Pol2(S2P) in Zscan4-spotty SN-type oocytes(top, *n* = 27/27), intense Pol2(S2P) in Zscan4-diffusive SN-type oocytes (middle, n = 2/2), intense Pol2(S2P) in Zscan4-diffusive NSN-type oocytes (bottom, *n* = 3/3). (*B*) SN- and NSN-type GV oocytes were immunostained for *Pol2(S5P)*: RNA polymerase II CTD repeat (phospholylated Ser5), a marker for active transcriptional initiation.

It has been shown that large-scale changes in nuclear organization are associated with modification of histone and chromatin-bound proteins (De La Fuente 2006). A previous study on Br-UTP incorporation in GV oocytes demonstrated that overall transcription level is high in NSN-type GV oocytes, whereas it is repressed in SN-type GV oocytes (Bouniol-Baly et al. 1999; De La Fuente and Eppig 2001; Miyara et al. 2003). Hence, we asked whether the Zscan4 immunostaining pattern observed in SN- and NSN-type oocytes is associated with transcriptional status by assessing phosphorylation of Ser2 in RNA polymerase II CTD repeat, Pol2(S2P), a marker for active transcriptional elongation of RNA polymerase II (Hsin and Manley 2012; Phatnani and Greenleaf 2006) (Fig. 5 *A*; Supplementary Movie 4). Remarkably, all of the NSN-type GV oocytes showed intense Pol2(S2P) immunostaining with multiple foci throughout the nucleus, indicating that RNA polymerase II-mediated transcriptional elongation is active in NSN-type GV oocytes. By contrast, Pol2(S2P) immunostaining was largely diminished in SN-type GV oocytes with spotty Zscan4 localization. Notably, SN-type GV oocytes with dispersed Zscan4 localization showed an intense Pol2(S2P) immunostaining pattern similar to NSN-type. Essentially, the same phenomena were observed in the immunostaining pattern of phosphorylation of Ser5 in RNA polymerase II CTD repeat Pol2(S5P), a marker for active transcriptional initiation (Phatnani and Greenleaf 2006) (Hsin and Manley 2012) (Fig. 5 *B*). Taken together, these results indicate that Zscan4 localization patterns are more precisely correlated with the transcriptional status of GV oocytes than DAPI-staining patterns, i.e., NSN or SN. Essentially, all GV oocytes with a spotty Zscan4 localization pattern are transcriptionally silent, whereas GV oocytes with a diffuse Zscan4 localization pattern are transcriptionally active. On the other hand, some of the SN-type GV oocytes—when Zscan4 shows a diffusive localization pattern—are transcriptionally active, despite the previous belief that the SN-type GV oocytes are transcriptionally silent.

### Zscan4 is expressed in late prophase spermatocytes and Sertoli cells

**Figure 6.**
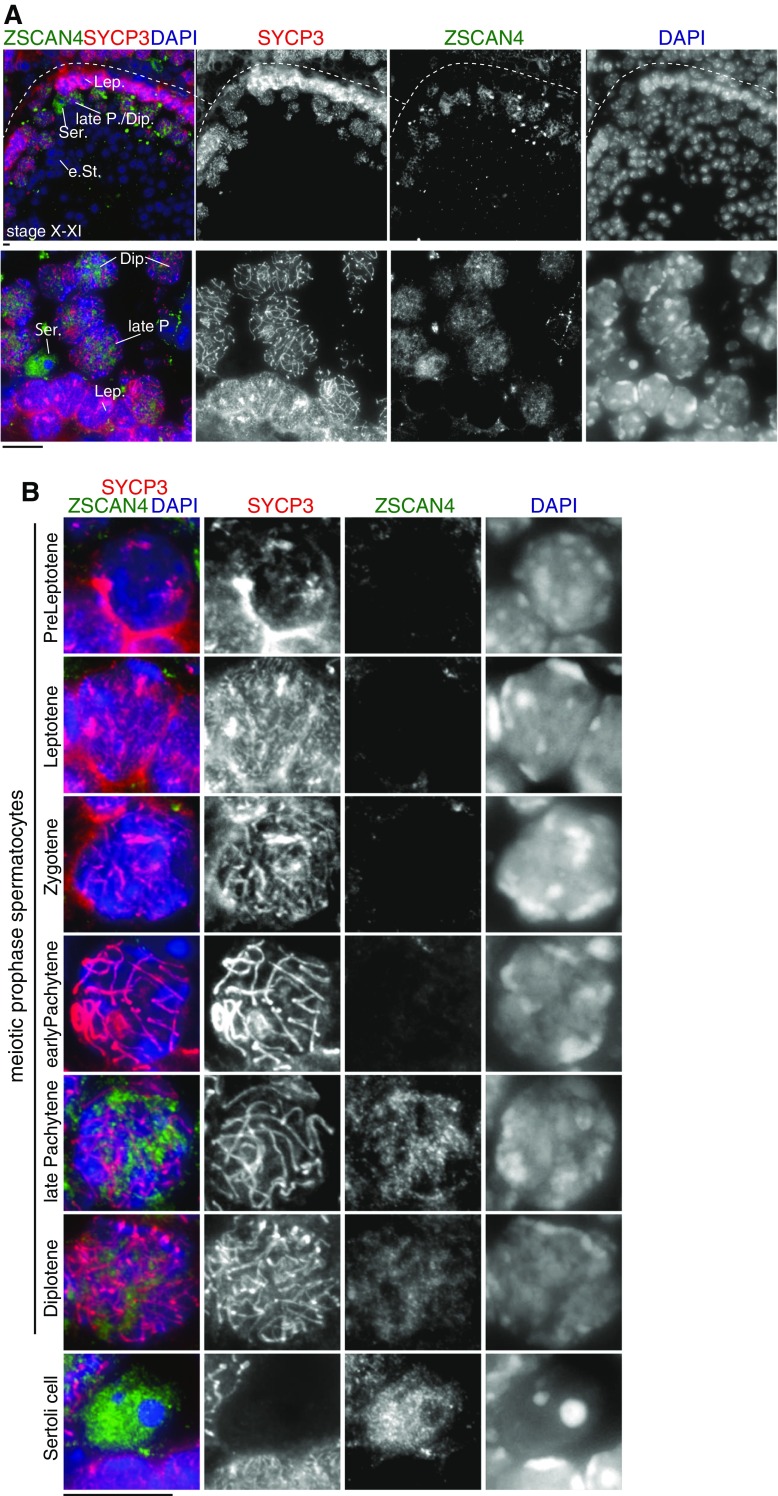
Zscan4 protein is expressed in late prophase spermatocytes and Sertoli cell. (*A*) Seminiferous tubule sections were immunostained as indicated. Stage X-XI tubule is shown (*upper*). Higher magnitude image is shown (*lower*). *Lep* leptotene, *late P.* late pachytene, *Dip* diplotene, *e St.* elongated spermatid, *Ser.* Sertoli cell. (*B*) Zscan4 immunostaining of spermatocytes at different stages of meiotic prophase and Sertoli cells are shown. *Scale bars*, 5 μm.

Because our RT-PCR suggests that *Zscan4* mRNA is modestly expressed in the adult testis (Fig. 1), we asked whether Zscan4 is expressed in any cell population in the testis at a protein level. Intriguingly, we noticed that Zscan4 was faintly but consistently immunostained in a subset of spermatocytes at particular stages of seminiferous tubule sections (Fig. 6 *A*). It is worth noting that Zscan4 was highly detectable only in late pachytene or diplotene spermatocytes within the stage X-XI seminiferous tubules, but not in spermatocytes at other stages, spermatogonia, or spermatids (Fig. 6 *B*). Contrary to this observation, embryonic testis sections did not show any detectable levels of Zscan4 immunostaining (Supplementary Fig. 3). These results suggest that Zscan4 is expressed in a very restricted timing—late pachytene or diplotene—during male meiosis, which is reminiscent of the restricted expression in late meiotic prophase in oocytes (Fig. 3).

Interestingly, significant levels of Zscan4 immunostaining were also detected in Sertoli cells, in which the signal was more intense compared to late pachytene/diplotene spermatocytes. It is known that Sertoli cells respond to retinoic acid (RA) and cyclically change their functions in a coordinated manner with spermatogenesis (Sugimoto et al. 2012); (Hasegawa and Saga 2012). Because the *Zscan4* locus can be activated in response to RA stimuli in mouse ES cells (Sharova et al. 2007; Sharova et al. 2016) (also, see accompanied manuscript), the expression of Zscan4 in Sertoli cells may be related to the periodic seminiferous cycle in response to RA.

## Discussion

In this study, we have shown for the first time that Zscan4 is expressed at a protein level not only in preimplantation embryos but also in specific stages of the post-natal ovary and testis. Notably, in both ovary and testis, Zscan4 is detectable in oocytes and spermatocytes during late meiotic prophase. Because meiotic prophase is equivalent to a prolonged G2 phase in mitotic cell cycle (Eichenlaub-Ritter 2012), the aforementioned data are reminiscent of the observation that Zscan4 is transiently activated at the G2 phase in ES cells (Storm et al. 2014); (Nakai-Futatsugi and Niwa 2016) and in the late two-cell stage (Falco et al. 2007). Although the precise roles of Zscan4 during meiosis are yet to be clarified, the implication for these results is that Zscan4 may play an important role in germ cell lineages as observed in ES cells (Zalzman et al. 2010; Amano et al. 2013; Akiyama et al. 2015).

Previously, we have shown that a burst of *Zscan4* transcription is accompanied by rapid derepression and rerepression of heterochromatin regions in mouse ES cells (Akiyama et al. 2015). Given that heterochromatic regions are reorganized into limited spots of clusters in mouse Zscan4-positive ESCs (Akiyama et al. 2015), our data suggest that similar mechanisms may play a role in heterochromatin organization in Zscan4-positive cell populations in reproductive organs. It is worth noting that Zscan4 is immunopositive in Sertoli cells, where heterochromatic regions are organized into an unusually large chromocenter (Fig. 6 *B*), reminiscent of the heterochromatin clustering in Zscan4-positive ESCs. Crucially, we demonstrated that distinct spatial localization patterns of Zscan4 in SN- and NSN-type GV oocytes are accompanied by the global heterochromatin organization around the nucleolus (Fig. 3 *A*), as shown that 3D genome architecture plays a crucial role in a panoply of cell functions (Krijger et al. 2016). Thus, it is plausible that Zscan4-positive status is associated with global heterochromatin organization in those cell types, though a possible causal relationship between Zscan4 expression and chromatin architecture awaits further investigation.

It has been elusive how SN rather than NSN oocytes shows higher developmental potential (Inoue et al. 2008; Monti et al. 2013), despite the fact that both of them can be ovulated after the hormonal peak. We have shown that two distinct patterns of Zscan4 immunostaining more precisely correlate with RNA polymerase II transcription status in GV oocytes (Fig. 5). Notably, we found that a minor fraction of SN-type GV oocytes shows a diffusive pattern of Zscan4 immunostaining (Fig. 5 *A*). It remains to clarify whether this minor fraction of GV oocytes represents intermediate status from NSN to SN transition (Mattson and Albertini 1990; De La Fuente 2006) or an intrinsic difference in the quality of cytoplasmic constitution (Monti et al. 2013).

Although multiple copies of highly identical *Zscan4* loci technically hamper genetic engineering of the endogenous locus, our recent success in generating a modified allele of *Zscan4* locus prompts us to pursue further genetic analysis. Thus, conditional disruption of either one or all of *Zscan4* loci will shed light on the further elucidation of Zscan4 functions in reproductive cell lineages in vivo*.*

## Conclusion

In ovaries, Zscan4 proteins were detected in germinal vesicle (GV) stage oocytes. Zscan4 showed different spatial localization patterns between SN and NSN oocytes, which correlated with RNA polymerase II-mediated transcriptional status. In testes, Zscan4 proteins were detected in spermatocytes at late pachytene/diplotene stages and in Sertoli cells. These results suggest that Zscan4 may play critical roles not only in preimplantation embryos but also in germ cell lineages in both males and females.

## Electronic supplementary material

Supplementary Figure 1 Immunostaining of M I and M II oocytes. (A) M I oocytes were immunostained with anti-mouse Zscan4 antibody. (B)Z-stack sections of Zscan4 immunofluorescence of MII oocytes obtained after in vitro maturation of SN (left) and NSN (right) antral oocytes, as indicated in (A). (GIF 107 kb)

High resolution image (EPS 3728 kb)

Supplementary Figure 2 Co-immunostaining of SN-type GV oocyte with different Zscan4 antibodies SN-type GV oocyte was immunostained with anti-mouse Zscan4 antibodies, which were raised in different species. Scale bar, 20μm. (GIF 50 kb)

High resolution image (EPS 2096 kb)

Supplementary Figure 3 Immunostaining of Zscan4 in embryonic testes. Embryonic testes from 13 - 18 dpc (E13-E18) were immunostained as indicated. (GIF 378 kb)

High resolution image (EPS 14252 kb)

Supplementary Figure 4 **3D reconstitution of immunostaining of Zscan4 in SN-type GV oocyte (PowerPoint file)** SN-type GV oocytes were immunostained for Zscan4. Z-section images were transformed into a 3D volume image rotating around the X axis. **3D reconstitution of immunostaining of Zscan4 in NSN-type GV oocyte (PowerPoint file)** NSN-type GV oocytes were immunostained for Zscan4. Z-section images were transformed into a 3D volume image rotating around the X axis. **3D reconstitution of co-immunostaining of Zscan4 and ACA in NSN-type GV oocyte (PowerPoint file)** SN-type GV oocytes were immunostained for Zscan4 and anti-centromere antigen (ACA). Z-section images were transformed into a 3D volume image rotating around the X axis. **3D reconstitution of co-immunostaining of Zscan4 and Pol2Ser2P in NSN- and SN-type GV oocyte (PowerPoint file)** NSN- and SN-type GV oocytes were immunostained for Zscan4 and RNA polymerase II CTD repeat (phospholylated Ser2). Z-section images were transformed into a 3D volume image rotating around the X axis. (PPTX 5352 kb)
