# Supplementary figures and images for: Zscan4 is expressed specifically during late meiotic prophase in both spermatogenesis and oogenesis
Source: In Vitro Cell Dev Biol Anim. 2016 Oct 3;53(2):167–78. doi: 10.1007/s11626-016-0096-z (PMC5311088; doi:10.1007/s11626-016-0096-z)

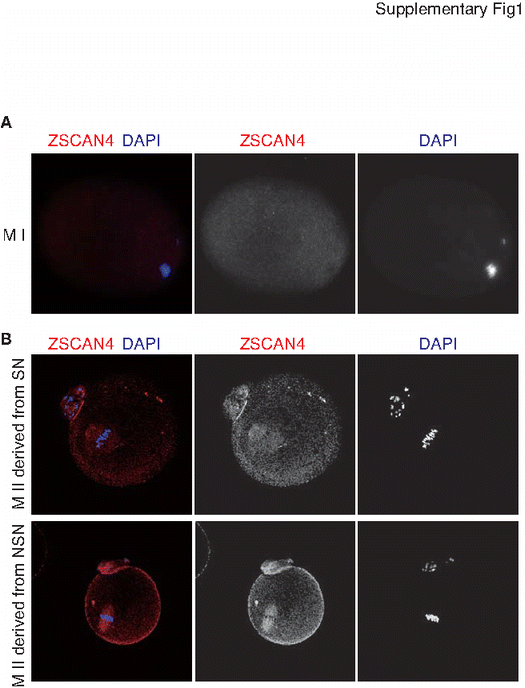

Supplement: Supplementary file 1 — Immunostaining of M I and M II oocytes. (A) M I oocytes were immunostained with anti-mouse Zscan4 antibody. (B)Z-stack sections of Zscan4 immunofluorescence of MII oocytes obtained after in vitro maturation of SN (left) and NSN (right) antral oocytes, as indicated in (A). (GIF 107 kb) [file 11626_2016_96_Fig7_ESM.gif]

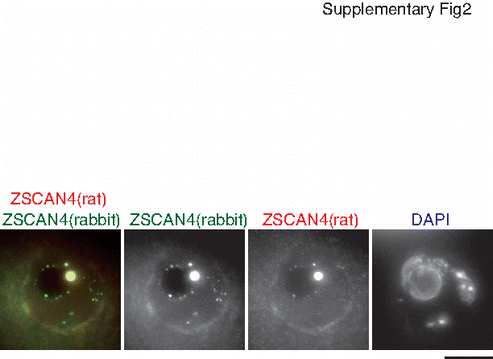

Supplement: Supplementary file 3 — Co-immunostaining of SN-type GV oocyte with different Zscan4 antibodies SN-type GV oocyte was immunostained with anti-mouse Zscan4 antibodies, which were raised in different species. Scale bar, 20μm. (GIF 50 kb) [file 11626_2016_96_Fig8_ESM.gif]

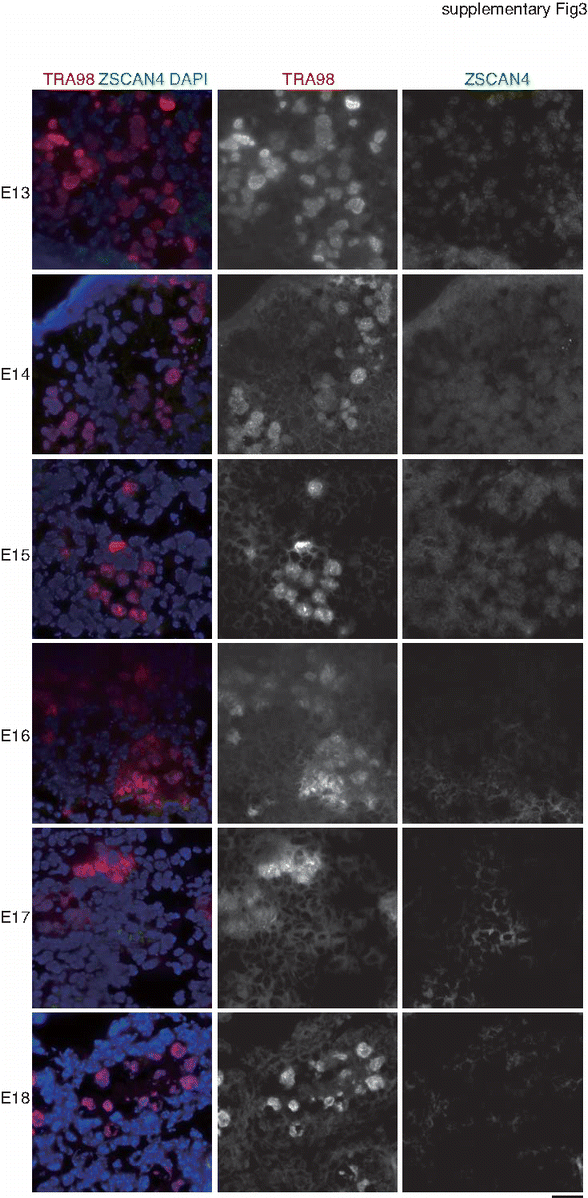

Supplement: Supplementary file 5 — Immunostaining of Zscan4 in embryonic testes. Embryonic testes from 13 - 18 dpc (E13-E18) were immunostained as indicated. (GIF 378 kb) [file 11626_2016_96_Fig9_ESM.gif]
