# Supplementary material for: Zscan4 is expressed specifically during late meiotic prophase in both spermatogenesis and oogenesis
Source: In Vitro Cell Dev Biol Anim. 2016 Oct 3;53(2):167–78. doi: 10.1007/s11626-016-0096-z (PMC5311088; doi:10.1007/s11626-016-0096-z)
Supplement: Supplementary file 7 — 3D reconstitution of immunostaining of Zscan4 in SN-type GV oocyte (PowerPoint file) SN-type GV oocytes were immunostained for Zscan4. Z-section images were transformed into a 3D volume image rotating around the X axis. 3D reconstitution of immunostaining of Zscan4 in NSN-type GV oocyte (PowerPoint file) NSN-type GV oocytes were immunostained for Zscan4. Z-section images were transformed into a 3D volume image rotating around the X axis. 3D reconstitution of co-immunostaining of Zscan4 and ACA in NSN-type GV oocyte (PowerPoint file) SN-type GV oocytes were immunostained for Zscan4 and anti-centromere antigen (ACA). Z-section images were transformed into a 3D volume image rotating around the X axis. 3D reconstitution of co-immunostaining of Zscan4 and Pol2Ser2P in NSN- and SN-type GV oocyte (PowerPoint file) NSN- and SN-type GV oocytes were immunostained for Zscan4 and RNA polymerase II CTD repeat (phospholylated Ser2). Z-section images were transformed into a 3D volume image rotating around the X axis. (PPTX 5352 kb) [file 11626_2016_96_MOESM4_ESM.pptx]

## Slide 1
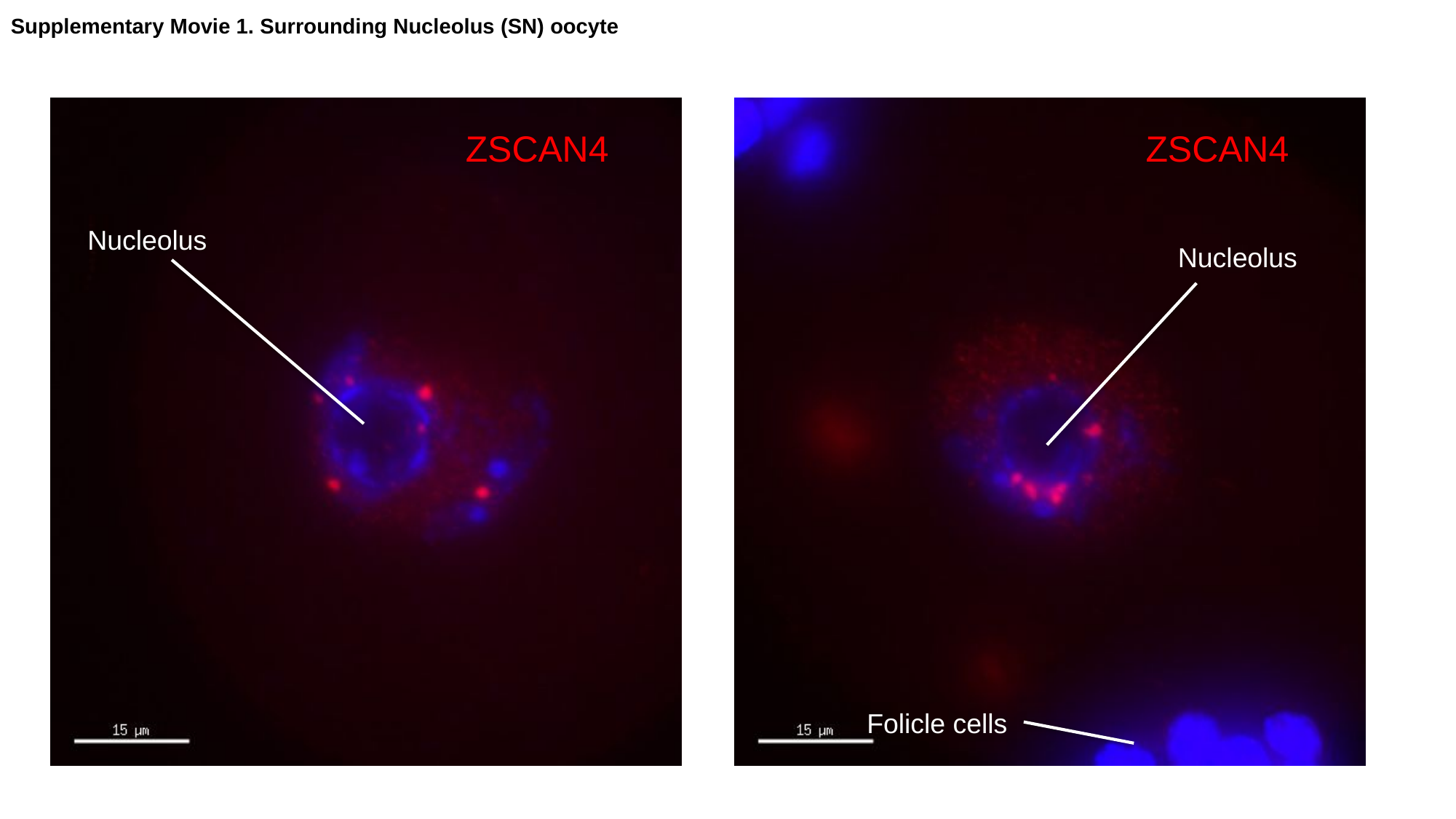

Supplementary Movie 1. Surrounding Nucleolus (SN) oocyte
ZSCAN4
ZSCAN4
Nucleolus
Nucleolus
Folicle cells

## Slide 2
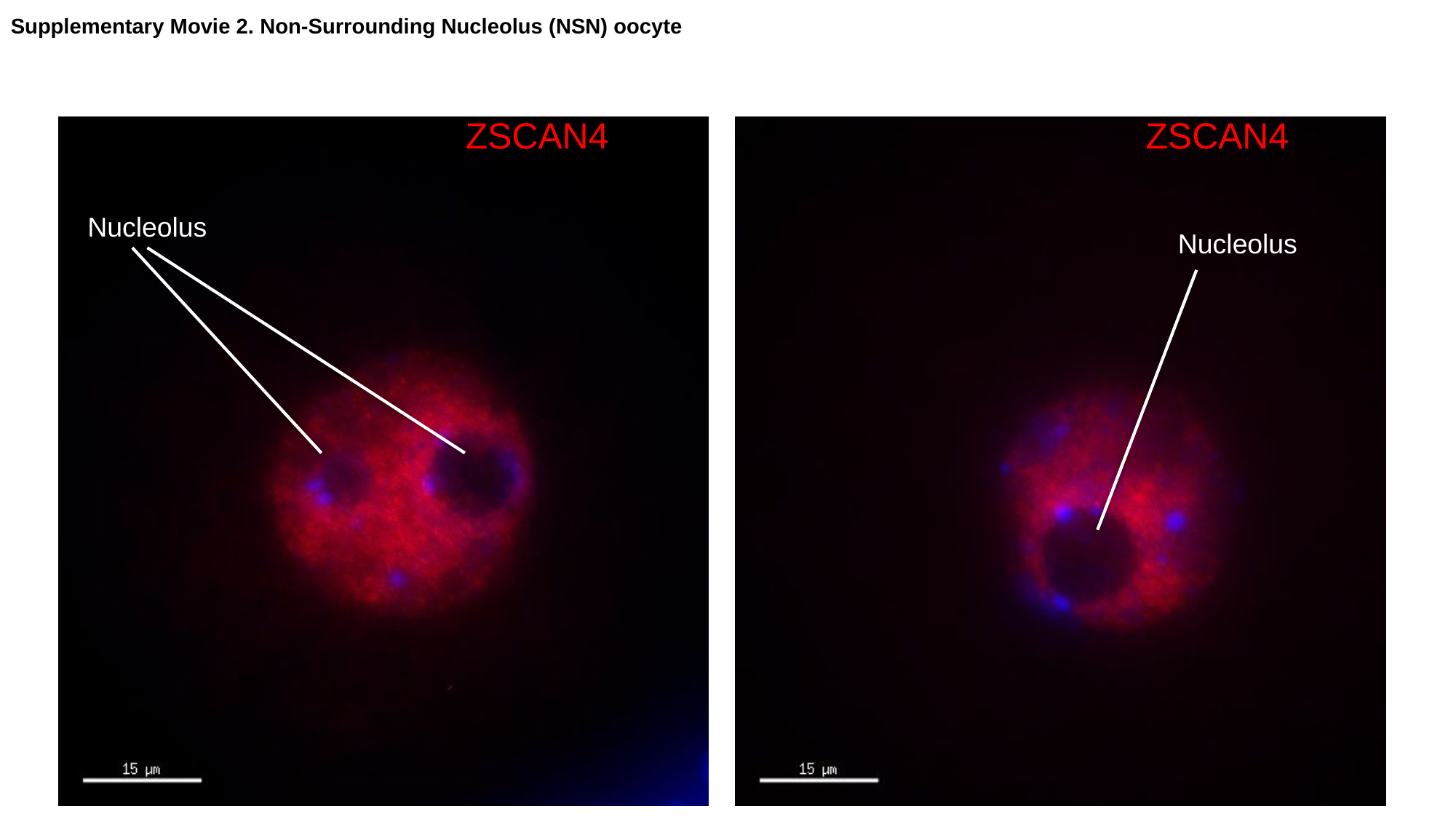

Supplementary Movie 2. Non-Surrounding Nucleolus (NSN) oocyte
ZSCAN4
ZSCAN4
Nucleolus
Nucleolus

## Slide 3
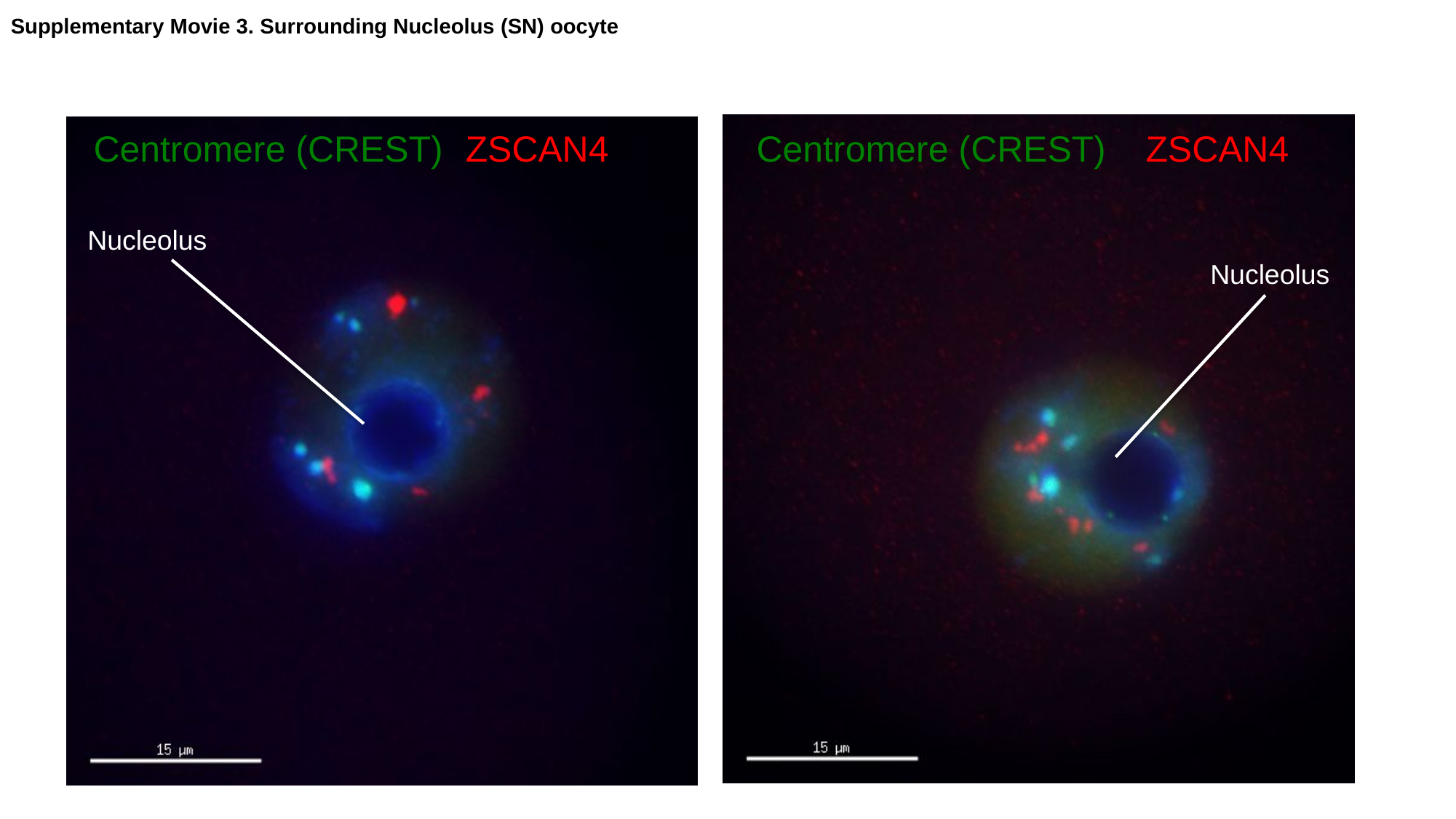

Supplementary Movie 3. Surrounding Nucleolus (SN) oocyte
Centromere (CREST)
ZSCAN4
Centromere (CREST)
ZSCAN4
Nucleolus
Nucleolus

## Slide 4
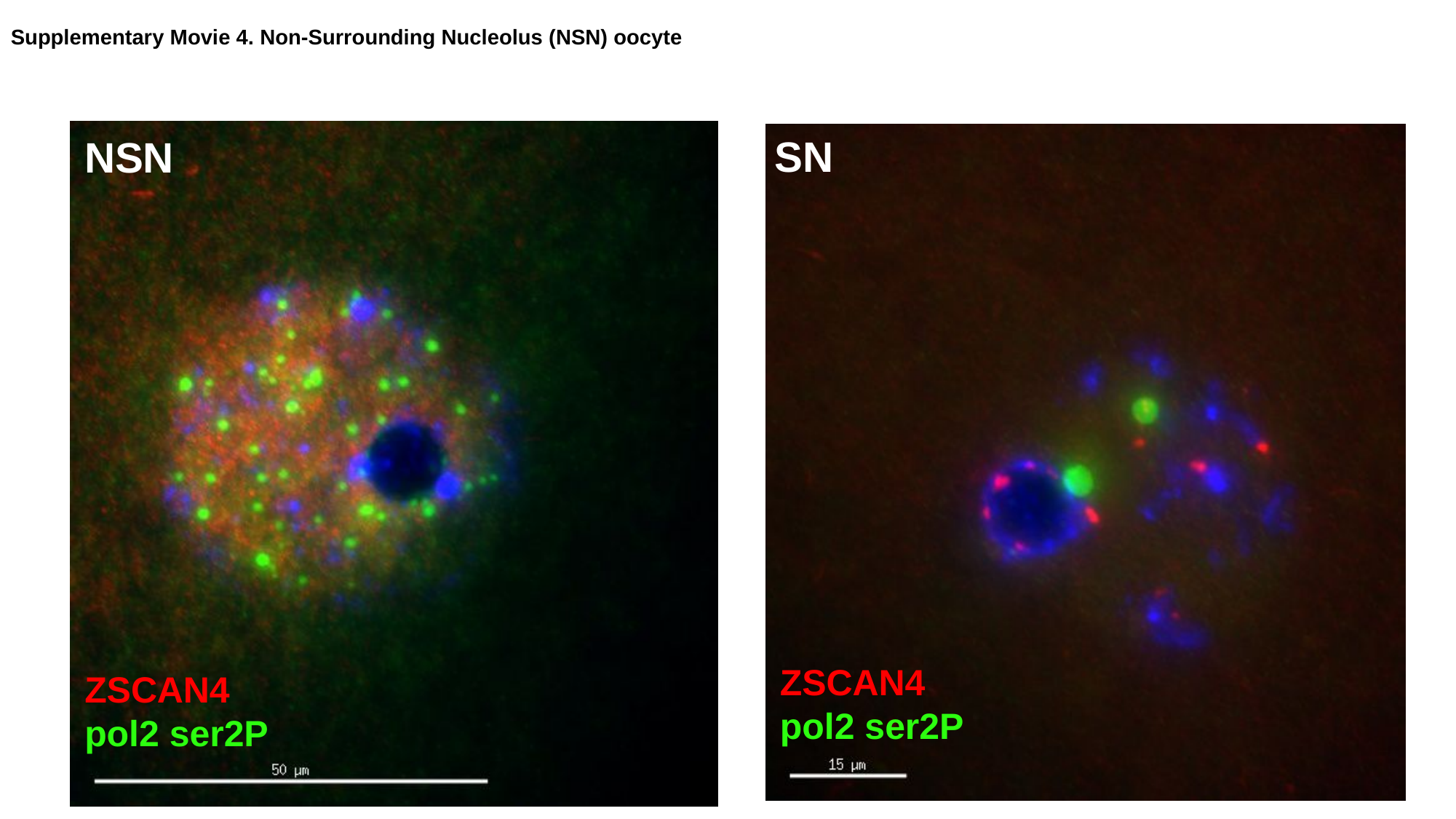

Supplementary Movie 4. Non-Surrounding Nucleolus (NSN) oocyte
SN
NSN
ZSCAN4
pol2 ser2P
ZSCAN4
pol2 ser2P
